# The Shepherds’ Tale: A Genome-Wide Study across 9 Dog Breeds Implicates Two Loci in the Regulation of Fructosamine Serum Concentration in Belgian Shepherds

**DOI:** 10.1371/journal.pone.0123173

**Published:** 2015-05-13

**Authors:** Simon K. G. Forsberg, Marcin Kierczak, Ingrid Ljungvall, Anne-Christine Merveille, Vassiliki Gouni, Maria Wiberg, Jakob Lundgren Willesen, Sofia Hanås, Anne-Sophie Lequarré, Louise Mejer Sørensen, Laurent Tiret, Kathleen McEntee, Eija Seppälä, Jørgen Koch, Géraldine Battaille, Hannes Lohi, Merete Fredholm, Valerie Chetboul, Jens Häggström, Örjan Carlborg, Kerstin Lindblad-Toh, Katja Höglund

**Affiliations:** 1 Computational Genetics Section, Department of Clinical Sciences, Swedish University of Agricultural Sciences, Uppsala, Sweden; 2 Science for Life Laboratory & Department of Medical Biochemistry and Microbiology, Uppsala University, Uppsala, Sweden; 3 Department of Clinical Sciences, Swedish University of Agricultural Sciences, Uppsala, Sweden; 4 Department of Clinical Sciences, Faculty of Veterinary Medicine, University of Liège, Liège Belgium; 5 Université Paris-Est, Ecole Nationale Vétérinaire d’Alfort, Unité de Cardiologie d’Alfort (UCA), Centre Hospitalier Universitaire Vétérinaire d’Alfort, Maisons-Alfort, France; 6 Department of Equine and Small Animal Medicine, Faculty of Veterinary Medicine, University of Helsinki, Helsinki, Finland; 7 Department of Veterinary Clinical and Animal Sciences, Faculty of Health and Medical Sciences, University of Copenhagen, Copenhagen, Denmark; 8 Evidensia, Animal Clinic Västerås, Västerås, Sweden; 9 INRA, UMR955 de Génétique Fonctionnelle et Médicale, Maisons-Alfort, France; 10 Université Paris-Est Créteil, CNM project, Ecole Nationale Vétérinaire d’Alfort, Maisons-Alfort, France; 11 Laboratory of Physiology, Faculty of Medicine, Université Libre de Bruxelles, Bruxelles, Belgium; 12 Department of Veterinary Biosciences, Research Program in Molecular Neurology Research Programs Unit, University of Helsinki, Helsinki, Finland; 13 Folkhälsan Institute of Genetics, Helsinki, Finland; 14 Department of Medical Genetics, University of Helsinki, Helsinki, Finland; 15 INSERM, U955, Equipe 03, Créteil, France; 16 Broad Institute of MIT and Harvard, Cambridge, MA, USA; 17 Department of Anatomy, Physiology and Biochemistry, Swedish University of Agricultural Sciences, Uppsala, Sweden; CSIRO, AUSTRALIA

## Abstract

Diabetes mellitus is a serious health problem in both dogs and humans. Certain dog breeds show high prevalence of the disease, whereas other breeds are at low risk. Fructosamine and glycated haemoglobin (HbA1c) are two major biomarkers of glycaemia, where serum concentrations reflect glucose turnover over the past few weeks to months. In this study, we searched for genetic factors influencing variation in serum fructosamine concentration in healthy dogs using data from nine dog breeds. Considering all breeds together, we did not find any genome-wide significant associations to fructosamine serum concentration. However, by performing breed-specific analyses we revealed an association on chromosome 3 (*p_corrected_* ≈ 1:68 × 10^-6^) in Belgian shepherd dogs of the Malinois subtype. The associated region and its close neighbourhood harbours interesting candidate genes such as *LETM1* and *GAPDH* that are important in glucose metabolism and have previously been implicated in the aetiology of diabetes mellitus. To further explore the genetics of this breed specificity, we screened the genome for reduced heterozygosity stretches private to the Belgian shepherd breed. This revealed a region with reduced heterozygosity that shows a statistically significant interaction (*p* = 0.025) with the association region on chromosome 3. This region also harbours some interesting candidate genes and regulatory regions but the exact mechanisms underlying the interaction are still unknown. Nevertheless, this finding provides a plausible explanation for breed-specific genetic effects for complex traits in dogs. Shepherd breeds are at low risk of developing diabetes mellitus. The findings in Belgian shepherds could be connected to a protective mechanism against the disease. Further insight into the regulation of glucose metabolism could improve diagnostic and therapeutic methods for diabetes mellitus.

## Introduction

Diabetes mellitus constitutes a major human public health problem [1] and is a growing problem also in dogs [2–4]. Diabetes mellitus is a group of metabolic diseases characterised by chronic excess of blood glucose (hyperglycaemia), as a result of defects in insulin secretion, insulin action, or both. Chronic hyperglycaemia is associated with long-term damage, dysfunction, and failure of various organs, especially the eyes, kidneys, nerves, heart, and blood vessels [1]. The hyperglycaemia leads to glycosuria, and affected individuals will show clinical signs including polyuria, polydipsia and weight loss despite increased appetite. Treatment often consists of daily insulin injections. In order to monitor glycaemic control, blood biomarkers can be measured. The main biomarker is HbA1c, a glycated haemoglobin. The lifespan of the erythrocyte is 100–120 days, and HbA1c reliably represents the glycemic control over the last 2–3 months. However, it cannot determine short-term changes in a patient’s glucose control [5]. A second biomarker of glycaemia is fructosamine, which is formed in a non-enzymatic, irreversible reaction between glucose and free amino groups on serum proteins [6]. The major blood protein, albumin, has a circulating lifespan of 15–20 days and fructosamine reflects the average blood sugar concentration over the past 2–3 weeks. It can therefore be used to determine more short-term changes in a patient’s glucose control [6].

The domestic dog (*Canis familiaris*) has been accompanying humans for several thousand years [7–9]. Modern dogs and humans share numerous common and complex diseases and have shared the same environment for many generations [9, 10]. Over the last 200 years, strong selection for certain traits have created dog breeds with unique diversity among mammalian species. However, during the breed-formation process, genetic variation within breeds became limited, leading to enrichment of some risk alleles and, as a consequence of this, to elevated rates of specific diseases. Due to the strong recent selection, canine genomes are characterised by relatively long haplotypes [9, 11, 12]. Together, these factors make the domestic dog a useful model for genetic studies of human complex diseases [10, 13, 14]. Of particular interest for this study is that some dog breeds show a high prevalence of diabetes mellitus (e.g., various terrier and spaniel breeds), whereas other breeds (e.g. German shepherds and Golden retrievers), have a very low prevalence of the disease [2, 15].

A EU-funded project with the acronym LUPA, focuses on investigating the genetic architecture underlying natural variation of various metabolic variables in healthy dogs [16]. The present study aimed to investigate the genetics underlying variation in fructosamine serum concentrations, using dogs examined within the project. We performed a series of genome-wide association studies for individual breeds as well as across breeds. We found a significant association to fructosamine concentration specific to the Belgian shepherd (BS) breed. Notably, the observed association was accompanied by a second nearly-fixed region. The results, thus, suggest a breed-specific genetic variability for this trait, potentially connected to a protective trait against development of diabetes mellitus in the BS breed. Further in-depth analyses are needed to fully understand the mechanisms involved in the genetic regulation of fructosamine concentration and glucose metabolism.

## Materials and Methods

### Animals

We examined privately-owned dogs coming from five centers participating in the EU-funded LUPA project [16]. At each center, dogs from 2–4 breeds were included. Some breeds were shared between centers and in total 9 breeds were included. The breeds were selected to represent variation in size, appearance, characteristics (shepherd, hunting dog, companion dog) of dogs in the best possible way. Apart from this, we also looked at the availability of individuals within breeds in the participating countries in order to reach desired sample size. In order to be included in the study, dogs had to be pure-bred, healthy, between one and seven years of age, and they had to have a normal body condition score. They could also not be related to each other at parental level, according to the pedigree for each dog. Each breed cohort included dogs of one sex only; females that were in anestrous or spayed, or intact males. Exclusion criteria consisted of any finding indicating systemic or organ-related disease observed at the clinical examination outlined below. In the Belgian shepherd breed, only dogs of the Malinois subtype were included. We performed examinations at University of Liège, Belgium; University of Copenhagen, Denmark; Alfort school of veterinary medicine, France; University of Helsinki, Finland and the Swedish University of Agricultural Sciences, Sweden.

### Ethics Statement

The study was carried out in accordance with the recommendations in the Guide for the Care and Use of Laboratory Animals of the National Institutes of Health and approved by an ethical Committee in Belgium (Commission d’Ethique Animale, Université de Liegè, Belgium, permit number 754), Sweden (Uppsala Local Ethical Committee, Uppsala, Sweden, permit number C 115/8), Finland (Viikki Campus Research Ethics Committee, Helsinki, Finland, no approval number used by committee) and Denmark (Local Ethical Committee, University Hospital for Companion Animals, Copenhagen, Denmark, at the time of the approval, the committee did not operate with approval numbers). In France, data was obtained before the creation of the Local Ethical Committee dedicated to clinical research (ComERC-ENVA). As the data were from client owned dogs undergoing normal veterinary exams, there was no animal experiment according to legal definitions in France. However, all local regulations related to clinical procedures were observed. In all countries, informed owners consent was obtained for use of samples and data for scientific research. All undertaken procedures were part of routine veterinary clinical exam in all countries and the local responsible co-investigator, who was also licensed to practice veterinary medicine within the EU, was responsible for collection of the samples.

### Sampling and verification of health status

On examination day, all dogs were fasted and without access to water for at least two hours before the examination. Each dog underwent a physical examination, blood pressure measurement by high-definition oscillometry, ECG recording and a standard echocardiographic examination, as part of the LUPA protocol.

Urine samples were collected by natural micturition and we performed standard urine analysis by dipstick and refractometer. We performed blood sampling by venipuncture and collected blood into 5-ml EDTA and serum tubes. In order to further verify the health status, we performed routine analysis of haematology and biochemistry including parameters of liver and kidney function, total serum protein concentration and serum electrolyte concentrations. Serum tubes for analysis of metabolic variables were centrifuged within 30 minutes of blood sample collection. Next, we harvested serum, transferred it into plastic cryotubes and froze it along with EDTA tubes for genetic analysis. Samples were kept at −80°C, later transported frozen to accredited laboratories (one for analysis of metabolic variables and one for genetic analyses), where they were analysed in batches by trained personnel.

### Analysis of metabolic variables

We analysed serum concentrations of glucose, insulin and fructosamine, using the following assays: glucose—Konelab 60i (Thermo Electron Co, Finland); insulin—RIA (DiaSorin S.p.A, Italy); fructosamine—Hariba ABX assay (Montpellier, France) analysed using Konelab 60i (Thermo Electron Co, Finland).

### Data

We collected samples from 501 healthy dogs representing 9 breeds: Belgian shepherd, Boxer, Cavalier King Charles spaniel, Dachshund, Doberman pinscher, Finnish lapphund, German shepherd, Labrador retriever and Newfoundland (Table 1).

**Table 1:** Overview of the breeds represented in the study.

**Breed**	**Abbreviation**	**Number of dogs (females, males)**
Belgian shepherd	BS	118 (0, 118)
Boxer	BOX	15 (0, 15)
Cavalier King Charles spaniel	CKCS	33 (0, 33)
Dachshund	DACH	40 (0, 40)
Doberman pinscher	DOB	22 (0, 22)
Finnish lapphund	FIN_LAP	45 (0, 45)
German shepherd	GS	65 (0, 65)
Labrador retriever	LAB	122 (72, 50)
Newfoundland	NF	41 (41, 0)
**Total**	—	**501 (113, 388)**

### Descriptive statistical analysis

We performed all analyses and visualisations using the R software framework for statistical computing and visualisation [17]. First, we used the Pearson’s *χ* ^2^ test to determine whether fructosamine concentrations significantly deviated from normality. Neither the overall distribution of fructosamine concentrations (Pearson’s *χ* ^2^ test; *p* = 0.89), nor the breed-specific distributions showed any significant departures from normality. Since serum fructosamine concentration is affected by total serum protein concentration [18], we analysed the correlation between the two using Pearson’s correlation coefficient *r*.

### Genotyping and quality control

All individuals have been genotyped using the Illumina 170k CanineHD Bead Chip (Illumina, San Diego, CA, USA) and we imported the raw data into GenABEL-native format [19] for further analyses.

Before proceeding to association analyses, we performed an iterative genotyping quality control (QC) procedure. The original dataset contained genotyping information for 185,295 SNP markers. We removed 33,004 (17.8%) of these as non-informative markers with minor allele frequency below 5% and 13,600 (7.3%) markers due to call rate below 0.95. Thus, after quality control, 139,092 (75.1%) markers remained for further genetic analysis.

Using 2,000 randomly selected markers, we calculated the average allelic-frequency-weighted identity-by-state (IBS) for all pairs of individuals in the population. All pairwise IBS values were below 0.95, thus we did not remove any individuals due to excessive relatedness. Next, we computed an IBS-based distance matrix using all autosomal SNPs that passed quality control. We then used the matrix to perform multidimensional scaling (MDS) in order to to visualise the genomic-kinships between individuals in two dimensions (S2 Fig).

### Genome-wide association analyses

We performed all genome-wide analyses using the GenABEL R-package, version 1.7-0 [19]. To determine whether there were indications of population stratification or cryptic relatedness, we examined the genomic inflation factor *λ* and visualised observed vs. expected distribution of p-values using quantile-quantile (QQ) plots. We computed *λ* by using the highest 75% observed and theoretical p-values, expected under the null hypothesis. When testing for genotype-phenotype associations, we started by performing a basic association scan, as implemented in the GenABEL function qtscore. If there were indications of p-value inflation, we fitted a linear mixed model using the polygenic function implemented in GenABEL. For the across-breed analysis, the observed population structure (S2 Fig) led us to only fit linear mixed models including a fixed effect for breed. We corrected for multiple testing by applying a conservative Bonferroni correction to a *p*_*raw*_ = 0.05 significance threshold. In addition, we performed a permutation test based on 10,000 randomised datasets to empirically derive the significance of the obtained results based on the particular populations analysed. If there were indications of p-value inflation, we performed the permutations on the GRAMMAR+-transformed mixed model residuals [20] to account for the population structure.

### Breed-specific analyses

In the breed-specific analyses, we analysed only breeds in which more than 50 individuals were included, i.e.: Labrador retrievers (N = 122), Belgian shepherds (N = 118) and German shepherds (N = 65). For the across-breed analyses, we used the same approach and thresholds as outlined in the preceding paragraphs. S1 Table summarises QC results for the three breeds separately.

### Linkage analyses

Subsequently, we evaluated the linkage disequilibrium (LD) structure in regions surrounding the strongest associated significant markers. LD was measured using *r* ^2^ and visualised using the LDheatmap function from the package LDheatmap [21]. Gene annotations in those regions were compiled from the UCSC Genome Browser using the canFam2, canFam3.1 and hg19 assemblies. We used Ensemble annotations and Broad Improved Canine Annotations [22]. When required, we used liftOver software [24] to map between the assemblies.

### Scan for allele frequency difference

To identify regions where the Belgian shepherd breed alone shows reduced heterozygosity, we performed an across-breed scan for extreme differences in allele frequency. To this end, for every SNP, we calculated the absolute difference in the allele frequency of an arbitrarily chosen reference allele between BS and the remaining breeds: *f*_*diff*_ = |*f*_*BS*_−*f*_*remainingbreeds*_|, where *f*_*BS*_ and *f*_*otherbreeds*_ are the allele frequencies in the Belgian shepherd breed and the remaining breeds respectively. We then evaluated the statistical significance of the observed differences using Fisher’s exact test to compare the observed reference-allele counts in BS and other breeds against respective allele counts under the null hypothesis (*H*_0_: *The two compared groups are drawn from the same population i.e. no difference in allele frequency*).

In addition to the allele frequency analyses outlined in the previous paragraph, we performed in-depth analyses of fixation on chromosome CFA5. We computed fixation index, *F*_*ST*_ for three different sets of populations: BS vs. all the remaining breeds, pooled BS and GS vs. all the remaining breeds as well as for pooled BS, GS and BOX vs. all the remaining breeds. Next, for each set, we averaged *F*_*ST*_ values over a sliding window of *n* = 21 markers (10 upstream and 10 downstream of the central marker). Window size has been chosen empirically, and provides a good tradeoff between noise level and signal dilution. Non averaged *F*_*ST*_ per SNP are shown in S10 Fig.

## Results

All included dogs had passed a general health examination without abnormal findings, we did not detect any ECG abnormalities, and all echocardiographic variables were within reference range. We did not detect any clinically relevant abnormalities in the urine analysis of included dogs. Furthermore, we did not detect any clinically relevant abnormalities in the haematological or serum biochemistry variables, including glucose, insulin, fructosamine and total serum protein concentrations. We did not find any significant correlation between protein and fructosamine concentrations (*r* ^2^ = 0.082, *p* = 0.074) and hence we did not further consider serum protein concentration in our analysis. The median fructosamine concentration for all dogs was 289 *μ*mol/l (inter-quartile range (IQR) 263–312 *μ*mol/l). The upper reference value for the method was a fructosamine concentration of 380 *μ*mol/l. The distribution by breed is shown in S1 Fig. As expected, after initial analyses, we found the study population to be highly genetically stratified (S2 Fig), with strata that closely corresponded to the included breeds. Some breeds were also further stratified in subpopulations.

### Genome-wide association analyses

**Fig 1:**
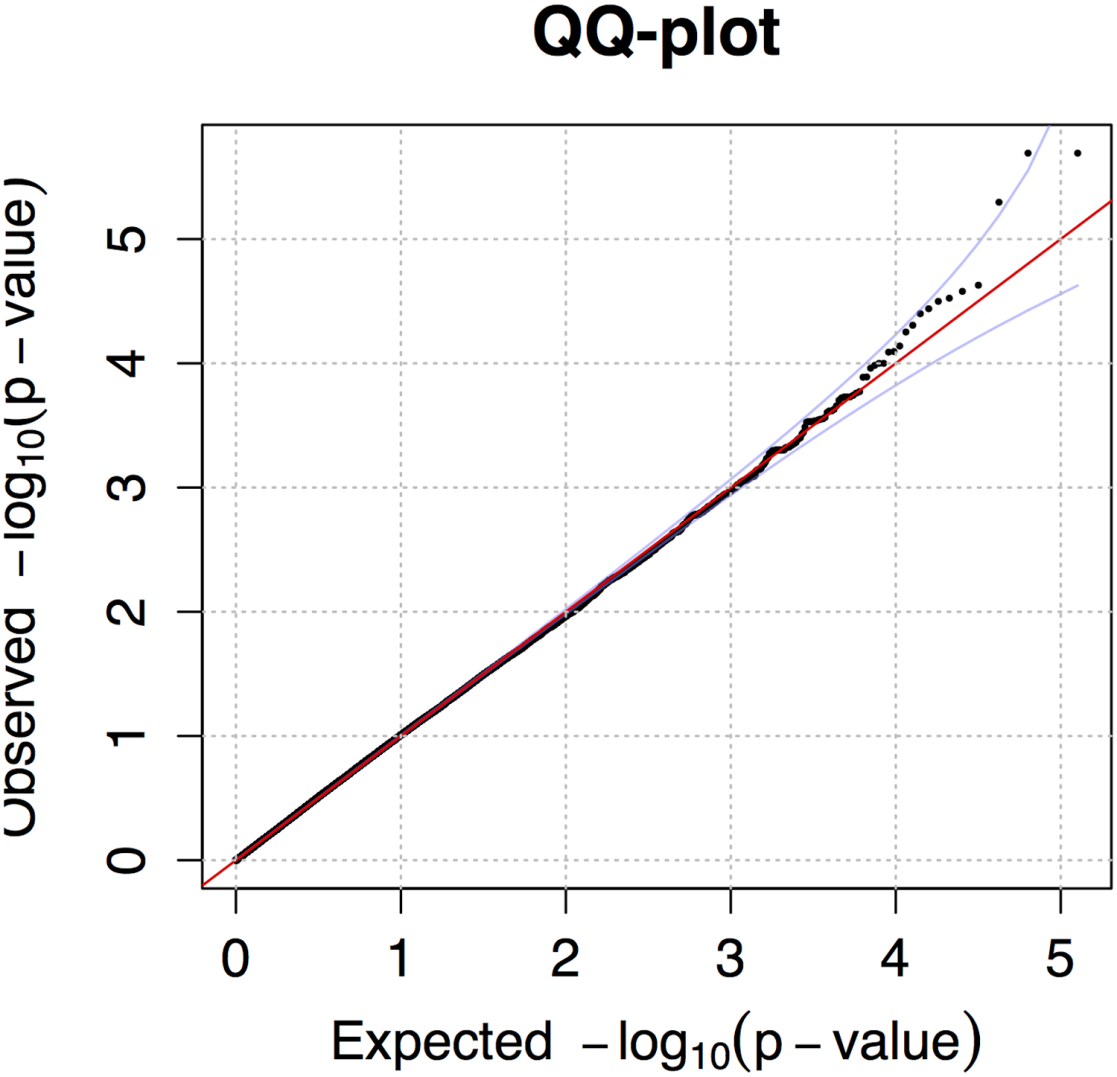
A QQ plot for the association to fructosamine concentration in Belgian shepherds. The plot corresponds to the Manhattan plot in Fig 2. Theoretical p-values are plotted against observed p-values (mixed model analysis). Blue lines denote 5% and 95% confidence intervals.

**Fig 2:**
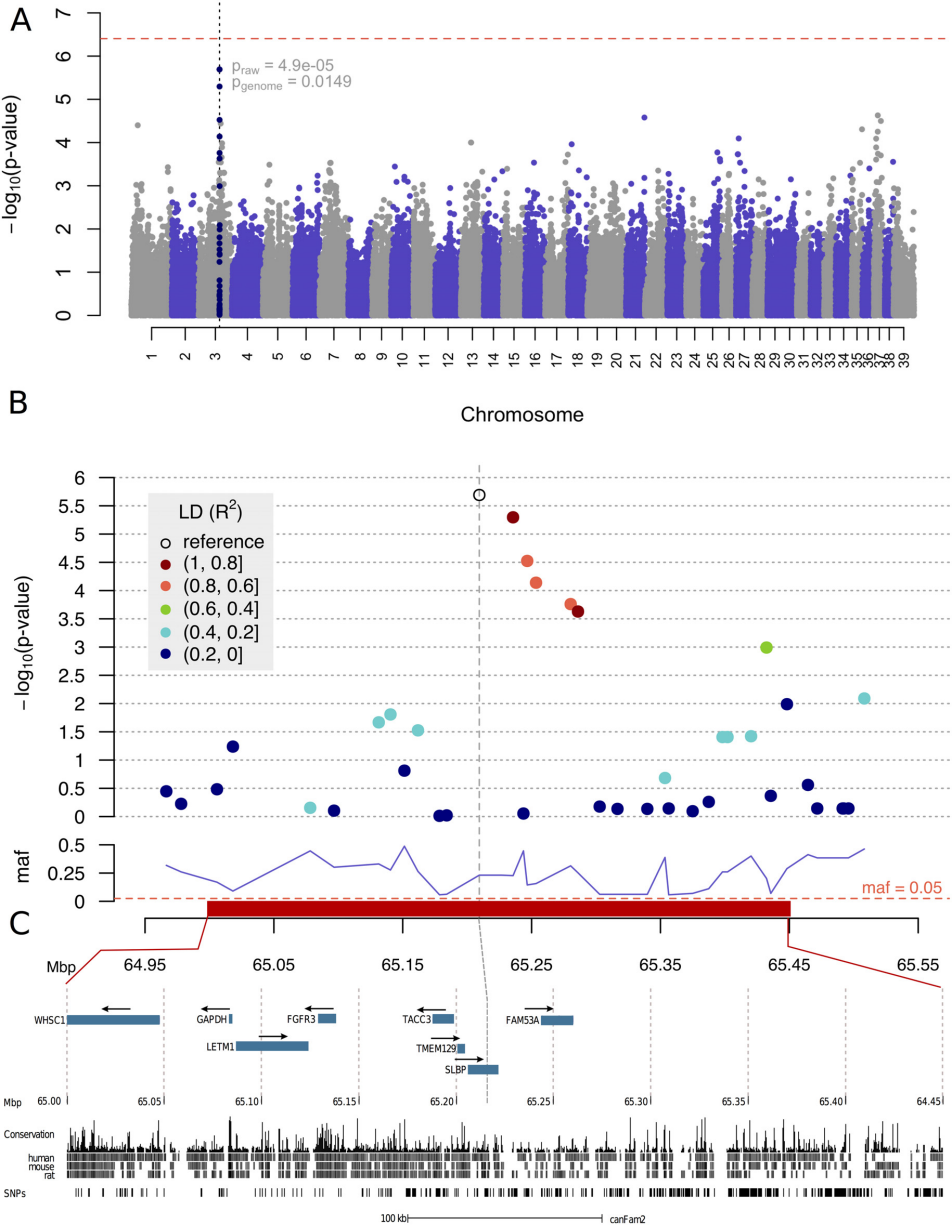
Manhattan plot showing association to fructosamine concentration in Belgian shepherds. Panel (A) shows a complete Manhattan plot with Bonferroni-corrected p-value of 0.05 marked as a red dashed line. Both raw and genome-wide p-values are given for the strongest associated marker. The association region is marked in navy blue and presented in more detail in panels (B) and (C). In panel (B) linkage disequilibrium (LD) to the top associated reference marker is shown and minor allele frequency is visualised below. Panel (C) shows details for a region on CFA3 between 64.95 and 65.25Mbp. Genes compiled from UCSC canine annotation, Broad Improved Canine Annotation v1 [22] and RefSeq [23] human homologue genes are represented by blue rectangles. Arrows show transcription direction. Also conservation across species (human, mouse, rat) is shown.

We performed a Genome Wide Association Study (GWAS) using fructosamine concentration as a continuous phenotype. When analysing all breeds together, we did not find any genome-wide significant associations to fructosamine concentrations (S4 and S5 Figs). In the breed-specific analyses, the standard GWAS analysis in BS (*N*_*BS*_ = 118) identified an association on chromosome 3 (FructoCFA3) that reached the genome wide Bonferroni-based significance threshold (0.05/*N*_*SNPs*_ ≈ 3.94 × 10^−7^, canFam2 *position*_*topSNP*_ = 65, 209, 415*bp*, Table 2). We interpreted the high genomic inflation value (*λ* ≈ 1.19) as an indication of potential cryptic relatedness in this breed and therefore also conducted a linear mixed model-based analysis incorporating the BS IBS-matrix to correct for this (Fig 1). The significance was lower in this analysis (*p*_*corrected*_ ≈ 1.68 × 10^−6^, Fig 2a) and no longer reached the Bonferroni correction-based threshold. Both results, however, exceed an empirical, genome-wide significance threshold obtained in a permutation test based on 10,000 randomised datasets (*p*_*perm*_ = 0.024). The standard GWAS results and permutation results are shown in S6 Fig. No genome-wide significant associations to fructosamine concentrations were found in any of the other breeds. Due to their significance for glucose metabolism, genome-wide association analyses for glucose and insulin concentrations were also performed in all breeds and within-breeds, however without finding any robust associations.

**Table 2:** Summary of identified loci. The table shows the leading SNPs at the two loci on CFA3 and CFA5.

**SNP ID**	**Chr**	**Position** (bp, canFam2)	**Alleles**	**Allele Frequency per breed** (%) (BS, GS, BOX, LAB, FIN_LAP, NF, DACH, CKCS, DOB)
BICF2S2344808	3	65209415	C/T	77, 99, 90, 60, 67, 9, 43, 84, 83
BICF2P1288638	5	66620365	G/G	1, 5, 0, 98, 88, 88, 91, 100, 78

### Linkage disequilibrium in the fructosamine associated region

The most associated SNP shows strong LD (*r* ^2^ > 0.8, S8 Fig) with 5 other SNPs encompassing a 100 kb haplotype (Fig 2). This haplotype contains the following genes: *transforming acidic coiled-coil containing protein 3* (*TACC3*), *transmembrane protein 129* (*TMEM129*), *stem-loop binding protein* (*SLBP*) and *family with sequence similarity 53* (*FAM53A*). Also, located ∼ 80kb from the leading SNP and outside the LD-block, we found the gene *fibroblast growth factor receptor 3* (*FGFR3*), ∼ 88*kb* from the leading SNP *leucine zipper-EF-hand containing transmembrane protein 1* (*LETM1*), ∼ 125*kb* from the most associated SNP (*r* ^2^ = 0.23) we found a highly similar (98% similarity to canine GAPDH) paralog of the gene *glyceraldehyde-3-phosphate dehydrogenase* (*GAPDH*) and ∼ 170*kb* from the most associated SNP, we found the *Wolf-Hirschhorn syndrome candidate 1* (*WHSC1*) gene.

### Epistasis and breed-specific effects

**Fig 3:**
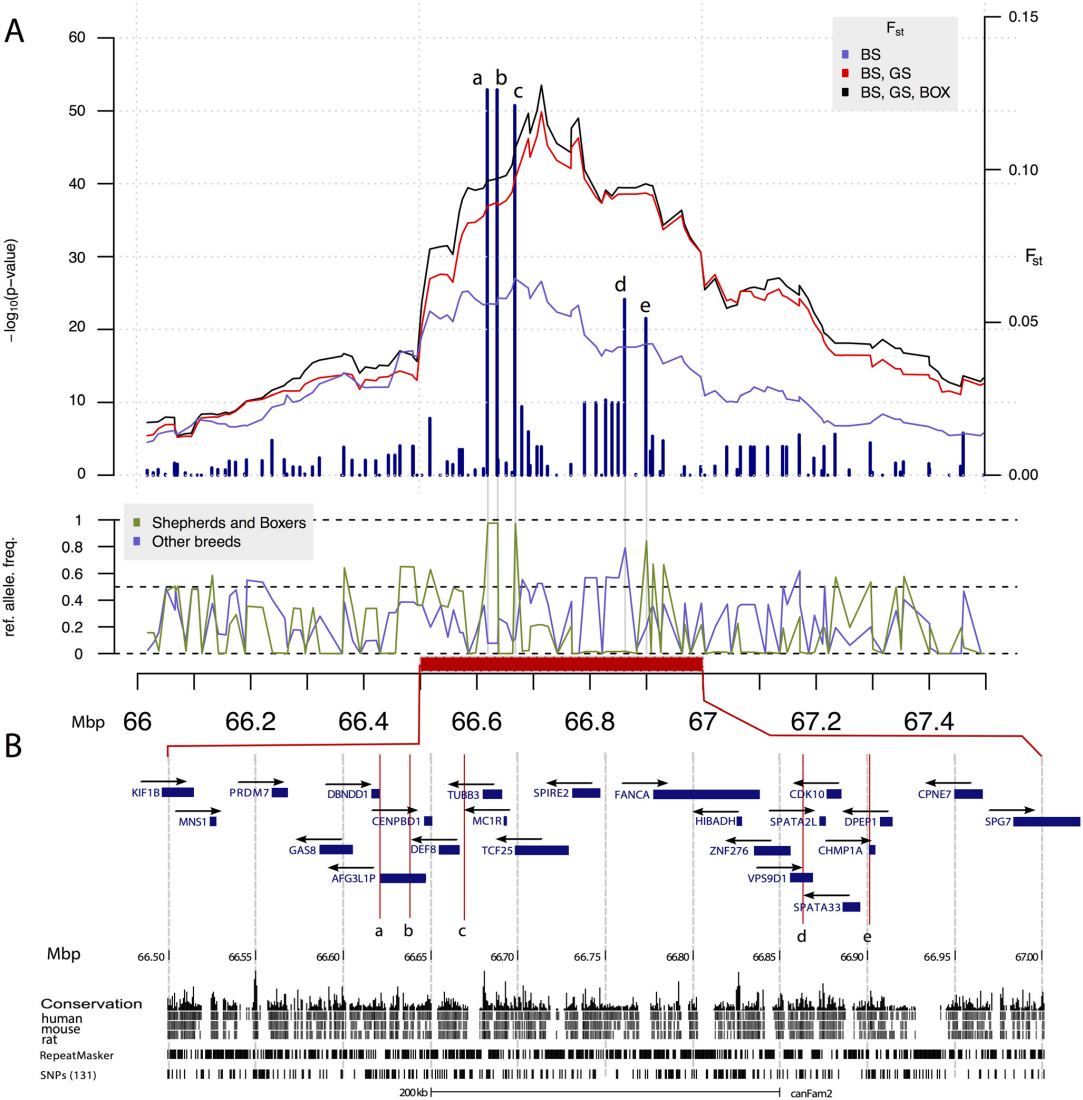
A reduced heterozygosity region on CFA5 specific to Belgian shepherds, German shepherds and Boxers. In panel (A), −*log*_10_(*p*−*value*) of the difference in reference allele count between Belgian shepherds, German shepherds and, Boxers vs. remaining breeds is presented. The three lines in the top panel show the *F*_*ST*_ values for three sets of comparisons: BS vs. all the remaining breeds (blue), pooled BS and GS vs. all the remaining breeds (red) as well as for pooled BS, GS and BOX vs. all the remaining breeds (black). The *F*_*ST*_ is averaged in a sliding window of 21 SNPs. The middle panel shows the allele frequencies per SNP for BS, GS, BOX (green) and the other breeds (blue). Panel (B) provides a detailed view on the most significantly differentiated region. Genes compiled from UCSC canine annotation, Broad Improved Canine Annotation v1 [22] and RefSeq [23] human homologue genes are represented by blue rectangles. Arrows show transcription direction. All annotation units likely to be pseudogenes were removed with the exception of AFG3L1P which overlaps with the strongest signal in the scan for allele frequency difference. Also conservation across species (human, mouse, rat) is shown. Letters on panel (A) mark loci with the lowest p-value and the same letters are used in panel (B) to denote position of the same markers.

To search for potential genetic determinants of the observed breed specificity, we performed an across-breed scan for extreme differences in allele frequency to identify regions where the BS breed alone shows reduced heterozygosity. Our working hypothesis was that such regions might contain alleles that modulate the effect of the FructoCFA3 locus through epistatic interactions. For every SNP, we calculated the absolute difference in an arbitrarily chosen reference allele frequency between BS and the remaining studied breeds *f*_*diff*_ = ∣*f*_*BS*_−*f*_*remainingbreeds*_∣, where *f*_*BS*_ and *f*_*otherbreeds*_ are the allele frequencies in the BS breed and the remaining breeds respectively. Using this method, we identified a locus on chromosome 5 (CFA5, Table 2, S9 Fig) that is close to fixation for one allele in BS. Upon closer examination, we found the identified locus to be close to fixation in BS, German shepherds and Boxers (*f*_*BS*, *GS*, *BOX*_ = 0.022, allele = G) (Fig 3), and almost fixed for the other allele in the remaining breeds (*f*_*otherbreeds*_ = 0.92, allele = A). We also performed additional analyses of the entire CFA5 comparing fixation index (*F*_*ST*_) between different subsets of BS, GS and BOX vs. the remaining breeds. The fixation index analyses results (Fig 3, S10 Fig) provide further evidence for the existence of a BS, GS and BOX-specific reduced heterozygosity region and also indicates that the observed pattern might be the signature of historical selection on this region. The detected region of reduced heterozygosity harbours several genes (including human RefSeq genes): *kinesin family member 1B* (*KIF1B*), *meiosis-specific nuclear structural 1* (*MNS1*), *PR domain containing 7* (*PRDM7*), *urate (hydroxyiso-) hydrolase* pseudogene (*URAHP*), *growth arrest-specific 8* (*GAS8*), *dysbindin domain containing 1* (*DBNDD1*), *AFG3-like AAA ATPase 1* pseudogene (*AFG3L1P*), *CENPB DNA-binding domains containing 1* (*CENPBD1*), *differentially expressed in FDCP 8 homolog* (*DEF8*), *tubulin, beta 3 class III* (*TUBB3*), *melanocortin 1 receptor* (*MC1R*), *transcription factor 25* (*TCF25*), *spire-type actin nucleation factor 2* (*SPIRE2*), *Fanconi anemia, complementation group A* (*FANCA*), *3-hydroxyisobutyrate dehydrogenase* (*HIBADH*), *zinc finger protein 276* (*ZNF276*), *VPS9 domain containing 1* (*VPS9D1*), *spermatogenesis associated 2-like* (*SPATA2L*), *cyclin-dependent kinase 10* (*CDK10*), *spermatogenesis associated 33* (*SPATA33*), *charged multivesicular body protein 1A* (*CHMP1A*), *renal dipeptidase 1* (*DPEP1*), *copine VII* (*CPNE7*) and *spastic paraplegia 7* (*SPG7*).

**Fig 4:**
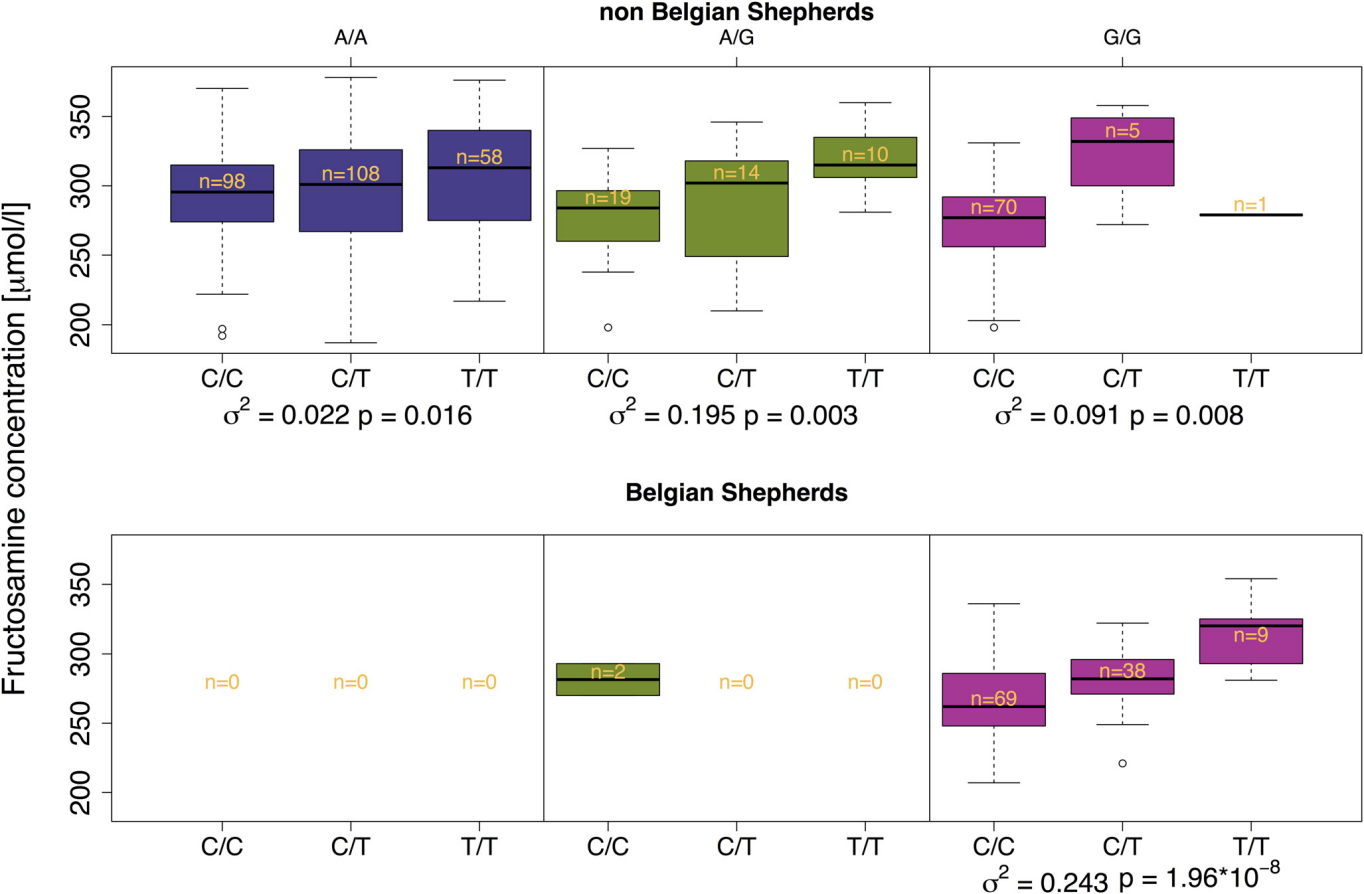
Two-locus genotype-phenotype map for FructoCFA3 and the CFA5 locus with reduced heterozygosity in the Belgian shepherd breed. The CFA5 interactor genotype is given above and the FructoCFA3 genotype below the figure. The top panel includes only non-BS dogs, and the bottom panel only BS dogs. The proportion of phenotypic variance explained by FructoCFA3 and the p-value of the correlation, in every strata defined by the interactor, are shown at the bottom. The number of individuals in every group are shown in the center of the boxes.

To evaluate whether this locus on CFA5 interacted with the main effect locus FructoCFA3, we fitted a linear regression model including both loci using the data from the non-Belgian shepherd populations, i.e. where the BS-allele on CFA5 segregated at a lower frequency and where FructoCFA3 did not have an effect in the earlier GWAS analyses. An F-test comparing the fit of a model with additive effects only for the two loci to a model including also the interaction between them shows that the interaction model provides a significantly better fit to the data (*p* = 0.025). To explore the epistatic genotype-phenotype relationship in more detail, we stratified the data by CFA5 genotype and estimated the effect of FructoCFA3 in each of the three genotype-classes independently (Fig 4). Despite the lower number of individuals in the A/G (*n* = 43) and G/G (*n* = 75) genotype classes, the results show that the effect of the FructoCFA3 locus is larger in the presence of the G-allele that is predominant in the BS breed.

We observed that the distribution of individuals within the G/G interactor class was highly skewed compared to the A/A and A/G classes (Fig 4). Using Pearson’s *χ* ^2^ test, we found the difference to be highly significant (*p* = 3.4 × 10^−16^), possibly suggesting a selection against the G/G (CFA5 locus) T/T (FructoCFA3) genotype combination. This signal is largely driven by GS dogs, since 64 out of the 65 GS dogs included in this study were homozygous at the CFA3 locus, having the C/C genotype.

## Discussion

Although the aetiology of diabetes mellitus is probably multifactorial, genetic factors may influence susceptibility [3, 25]. Diabetes mellitus is more common in certain dog breeds, such as terrier and spaniel breeds. Interestingly, certain dog breeds seem to have a decreased risk for developing diabetes [2, 3, 26], which could be due to protective alleles.

Several studies have reported a low prevalence of diabetes mellitus in the German shepherd breed [2, 27, 28]. The BS breed is related to the German shepherd and has in one study been shown to have a relatively low risk of diabetes mellitus [2]. The BS is a hard-working herding type of breed. Dogs are dependent on aerobic muscle metabolism when working [29]. During strenuous work, it is important to maintain an even blood sugar concentration in order to ensure energy supplies to the muscle cells. Potentially, during development of the BS breed, well-performing working dogs with a good blood sugar control might have been selected, thereby creating a breed with a protective trait against development of diabetes mellitus. However, this working hypothesis needs further investigation.

Since fructosamine is formed in a non-enzymatic, irreversible reaction between glucose and free amino groups on serum proteins [6], serum concentrations are affected not only by the average blood sugar concentration over the past 2–3 weeks, but also by lifespan and composition of serum proteins [30]. All dogs in the present study were healthy and their total serum protein concentration was within reference values. Furthermore, no correlation between fructosamine and serum protein concentration was found in the present study. Therefore fructosamine concentrations should reliably reflect the average blood sugar concentration of investigated dogs. While no influence of sex or age on fructosamine concentration has been shown in dogs [31], since our BS study population consisted of only males, we cannot be entirely sure that the association we found is not male-specific.

This study provides evidence that a particular locus might be correlated to a trait in some breed(s) while not in others. A possible explanation for such observations is that the causal variant(s), linked to the SNP(s) detected in the GWAS, might be present in one breed or population while not in others. Another possibility is that the different genetic backgrounds of the breeds influence the causal variant, making its effect penetrant only in some of them. We hypothesise that the detected interaction between the main effect locus FructoCFA3 and the chromosome 5 (CFA5) locus explains why FructoCFA3 only shows a significant effect in the BS breed. The interacting locus on CFA5 then acts as a capacitor of the allele at FructoCFA3, as illustrated in Fig 4. The detected association in BS then results from the fact that this particular breed is nearly fixed for the capacitor (G/G) genotype at this locus. As only non-BS dogs are included in the analyses in Fig 4, it provides independent evidence that dogs carrying the G/G or A/G interactor genotype display a stronger phenotypic correlation to FructoCFA3 across breeds. It is also worth noticing that this association explains a substantial amount of the phenotypic variance in the groups of individuals carrying the G/G (9.1%) or A/G (19.5%) interactor genotype, implying a major effect of the locus that might be of predictive value in the clinic.

Following our epistatic hypothesis, one would expect FructoCFA3 to show an effect also in the German shepherd and Boxer breeds, since the G allele at the interacting locus is also fixed, or very close to fixation, in these breeds. In the Boxers we do, despite the small sample size (n = 15), observe a nominally significant (p = 0.0019) association to fructosamine levels at the CFA3 locus which agrees with the epistatic pattern we observe between these two loci. The lack of association in German shepherds is explained by the fact that all but one of the German shepherd dogs carry the low fructosamine FructoCFA3 genotype C/C. The high fructosamine FructoCFA3 genotype T/T is apparently rarely observed in combination with the G/G interactor genotype. This trend can also be observed in Fig 4, where the distribution of individuals is skewed towards the low fructosamine FructoCFA3 genotype in the group carrying the G/G interactor genotype, compared to the other two groups. In this group, 70 out of 76 (92%) individuals carry the C/C low fructosamine FructoCFA3 genotype, compared to a more even distribution of individuals in the other two groups. This observation is interesting, since it might indicate a combined selection on the FructoCFA3 and CFA5 loci. Another possibility is that, by selecting only healthy dogs for this study, those with the G/G (CFA5 locus) T/T (FructoCFA3) genotype combination were excluded. We compared the distribution of individuals in the G/G interactor group to the A/A and A/G groups, using Pearson’s *χ* ^2^ test. This showed a highly significant difference (*p* = 3.4 × 10^−16^), supporting the observation that the high fructosamine FructoCFA3 genotype seem to be selected against, when in combination with the G/G interactor genotype.

Glycolysis, citric acid cycle and oxidative phosphorylation play central roles in the metabolism of most cell types and are crucial to glucose-induced insulin secretion from pancreatic *β*-cells [32]. In the glycolysis, glucose is broken down to pyruvate through a chain of chemical reactions in the cytosol. During the sixth step of glycolysis, nicotinamide adenine dinucleotide (*NADH*), which acts as a coenzyme in redox reactions, is generated. This step is catalysed by glyceraldehyde-3-phosphate dehydrogenase (*GAPDH*). A highly similar (98% similarity to canine *GAPDH*) *GAPDH*-paralog is localised approximately 125 kb from the main effect locus FructoCFA3. The *NADH* shuttle system transfers NAD+, the oxidized form of *NADH*, into the inner matrix of the mitochondria, the location for the citric acid cycle. Mitochondria consist of two membranes, a smooth outer membrane and a greatly convoluted inner membrane. The gene controlling Leucine Zipper-EF-Hand Containing Transmembrane Protein 1 (*LETM1*), localised approximately 88 kb from FructoCFA3, encodes a mitochondrial Ca2+/H+ antiporter on the inner mitochondrial membrane [33, 34]. Sufficient mitochondrial matrix calcium concentration is pivotal to trigger insulin secretion from the *β*-cell and experiments on *LETM1* knock-out mice have shown altered glucose metabolism in surviving heterozygous mice [34]. In a recent study [35], *LETM1* was shown to be down-regulated in obese mice, and it was suggested that it may participate in impaired insulin signalling in the adipose tissue. Other factors necessary to generate mitochondrial signaling leading to insulin secretion are being investigated, one candidate factor being *NADH* [32]. The *NADH* shuttles contribute to a sufficient amount of ATP being generated to trigger glucose-induced insulin secretion. Failure to generate mitochondrial metabolic signals through *NADH* shuttles might contribute to impairment of glucose-induced insulin secretion seen in diabetes mellitus [32].

The four genes within the haplotype the most tightly linked (*r* ^2^ to the most associated SNP > 0.8) to the FructoCFA3 signal are: *TACC3*, *TMEM129*, *SLBP* and *FAM53A*. Little is known about the potential link between glucose concentration and any of these genes.

Interestingly, the reduced heterozygosity region private to the shepherd breeds and Boxers on CFA5 harbours the melanocortin receptor *MC1R*. A dominant allele of this gene is known to cause the melanistic mask phenotype [36], a common phenotype in German shepherds, BS and Boxers but rare in the other breeds in our study. Indeed, we observe that all of these three breeds share the same pattern of reduced heterozygosity in this region, as opposed to the other breeds. It, thus, seems likely that the sweep-like pattern we observe is due to selection for the melanistic mask phenotype in these three breeds. This observation is further supported by the high *F*_*ST*_ values in this region (Fig 3). The melanocortin receptor pathway has been linked to several metabolic disorders including diabetes mellitus, obesity and hypoglycaemia [37–39]. However, to our knowledge, the *MC1R* subtype of the receptor family has not previously been linked to any metabolic phenotypes. It is thus unclear why we observe an epistatic effect on fructosamine concentrations, involving this locus. Either it has a so far unknown metabolic function, similar to other receptors in the family, or, even more likely, another allele has been co-selected with the the *MC1R* allele.

The reduced heterozygosity region on CFA5 harbours several other genes (Fig 3) that might play a role in the observed interaction. The role of AFG3-like AAA ATPase 1 pseudogene (*AFG3L1P*) that overlaps with the strongest association signal remains to be characterised. The charged multivesicular body protein 1A (*CHMP1A*) gene located in the vicinity of the 5-th most significant signal in the scan for allele frequency difference is a particularly interesting candidate gene that has been implicated in diabetes mellitus. In their recent study, Liu et al. [40] used network theory to—by combining epigenetic characteristics with human interactome—detect the potential interplay modules of DNA methylation and chromatin modifications specific for type 2 diabetes. Their results indicated that the aberrant DNA methylation pattern of *CHMP1A* gene might result in the disorders of epigenetic modules specific to type 2 diabetes.

### Conclusions

We detected an association to serum fructosamine concentration on chromosome 3 (CFA3), specific to the BS breed. Further analysis revealed a reduced heterozygosity region private to Boxers and the shepherd breeds on CFA5. This region was found to interact with the main effect region on CFA3, possibly explaining the lack of association in the non BS breeds. The nature of the potential interaction between FructoCFA3 and the reduced heterozygosity region on CFA5 is intriguing and will require further investigation. This interaction also presents a plausible hypothesis that might explain the lack of reproducibility of GWAS results across breeds for other traits as well. *GAPDH* and *LETM1* are both potential candidate genes that have been previously indicated both in glucose metabolism and impairment of insulin signalling. The results presented here might be a starting point for future studies, giving further insights into the susceptibility to diabetes mellitus. Further studies are however required to identify the causative genetic variants influencing fructosamine concentration.

## Supporting Information

S1 Fig Boxplots showing distribution of fructosamine by breed. The top, bottom and line through the middle of each box correspond to the 75th percentile (top quartile), the 25th percentile (bottom quartile) and the 50th percentile (median), respectively. The whiskers extend from the bottom 2.5th percentile to the top 97.5th percentile. Outliers, which are represented by black circles were included in the statistical analyses.
(PNG)

S2 Fig MDS-plot showing a two dimensional visualization of the genomic kinship between the individuals in the study population. The color indicates the breed and the symbol the country of origin. The plot includes 528 genotyped dogs, out of which 501 had serum fructosamine concentration measured.
(JPEG)

S3 Fig Two dimensional MDS-plot of the genomic kinship in the Belgian shepherd breed. (PNG)

S4 Fig A Manhattan plot visualization of the results from the mixed model association scan, including all breeds. No genome wide significant associations were found in this setup.
(PNG)

S5 Fig Quantile-quantile plot showing the p-values from the mixed model association scan including all breeds (y-axis) versus the theoretical p-values expected under the null hypothesis (x-axis). The red line shows the expected profile under the null (slope = 1).
(PNG)

S6 Fig A Manhattan plot visualization of the non-corrected association p-values in Belgian shepherds (top panel). The lower panel shows the p-values from the 10k permutation test, using the GRAMMAR+ transformed mixed model residuals.
(PNG)

S7 Fig The raw p-values from the non-corrected association scan in Belgian shepherds. The red line shows the profile expected under the null hypothesis (slope = 1) and the observed profile is indicated by the black line (slope = 1.19).
(PNG)

S8 Fig Heatmaps illustrating the pairwise LD between SNPs in Belgian shepherds. Panel (A) shows a 0.5 Mb segment surrounding the associated region on CFA 3. The leading SNPs in the GWAS analysis are indicated in blue. Panel (B) shows the region of reduced heterozygosity on CFA5. Indicated in blue are the SNPs showing the greatest difference in reference allele count when comparing Belgian and German shepherds vs. remaining breeds.
(PNG)

S9 Fig Histogram showing the genome-wide distribution of differences in allele frequencies when comparing Belgian shepherds to the other breeds (*f*_*diff*_ = ∣*f*_*BS*_−*f*_*remainingbreeds*_∣). The difference at the leading SNP in the CFA5 locus is highlighted in red.
(PNG)

S10 Fig Chromosome CFA5, fixation index (*F*_*ST*_) values per SNP, computed for BS vs. the remaining breeds, pooled BS and GS vs. the remaining breeds as well as for pooled BS, GS and BOX vs. the remaining breeds. There is a clear divergence between all three breeds (also in all pools) and all other breeds between 66–67Mb. Belgian shepherds alone show also high divergence around 81Mbp. The results are consistent with our analyses of allele frequency differences.
(PDF)

S1 Table Quality control summary. The table shows the number of markers removed due to low minor allele frequency (row 1) and low call rate (row 2). The first column shows the quality control results when analyzing all breeds together, and successive columns display the results from the breed specific analysis.
(PDF)
