# Supplementary figures and images for: The Shepherds’ Tale: A Genome-Wide Study across 9 Dog Breeds Implicates Two Loci in the Regulation of Fructosamine Serum Concentration in Belgian Shepherds
Source: PLoS One. 2015 May 13;10(5):e0123173. doi: 10.1371/journal.pone.0123173 (PMC4430432; doi:10.1371/journal.pone.0123173)

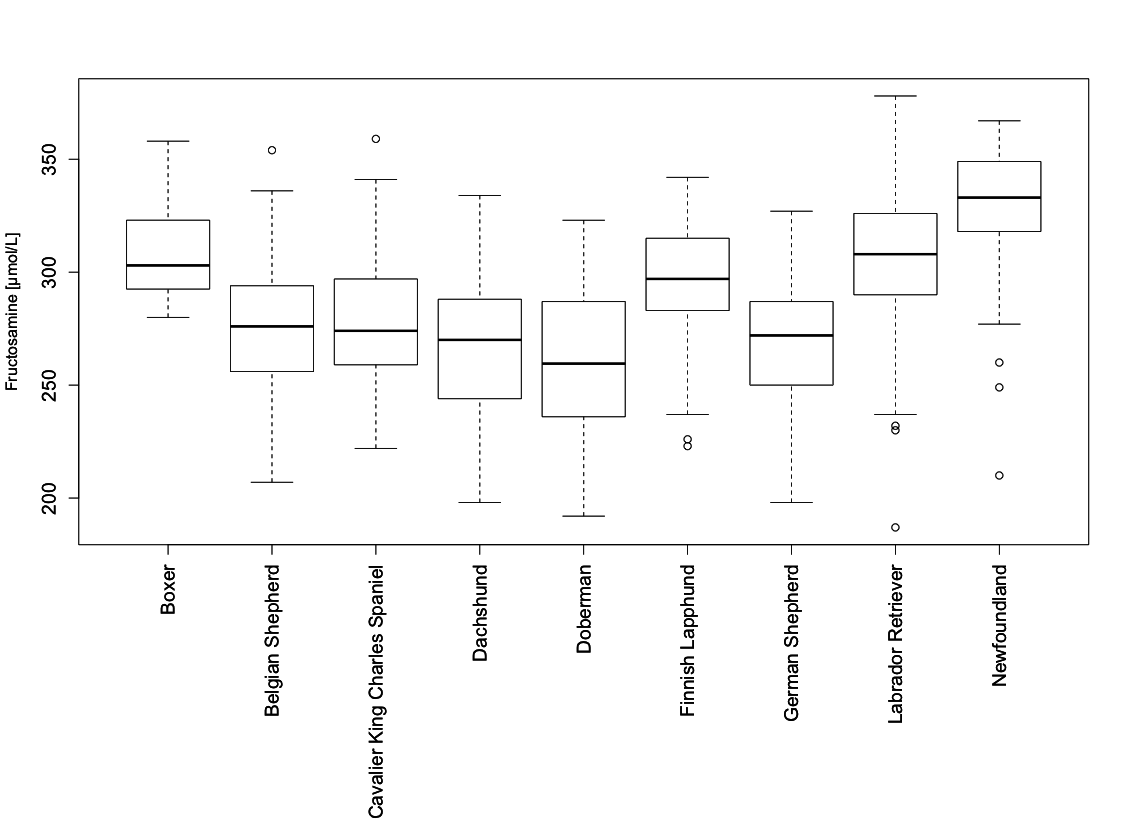

Supplement: S1 Fig — The top, bottom and line through the middle of each box correspond to the 75th percentile (top quartile), the 25th percentile (bottom quartile) and the 50th percentile (median), respectively. The whiskers extend from the bottom 2.5th percentile to the top 97.5th percentile. Outliers, which are represented by black circles were included in the statistical analyses. (PNG) [file pone.0123173.s001.png]

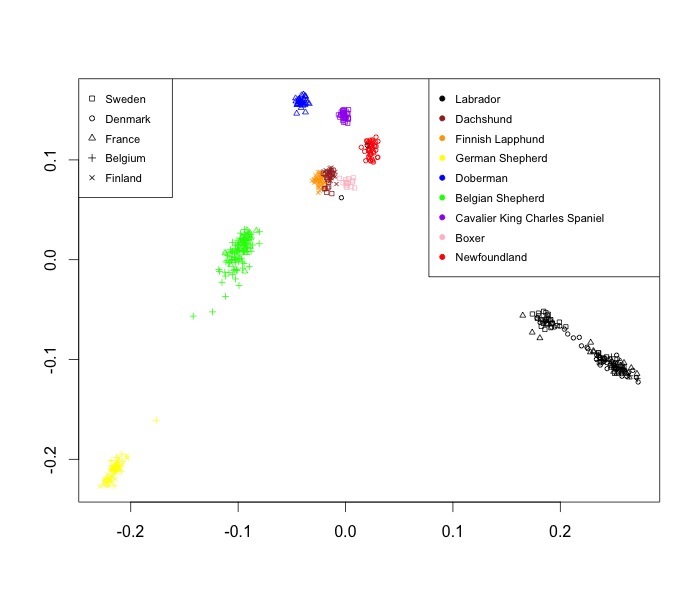

Supplement: S2 Fig — The color indicates the breed and the symbol the country of origin. The plot includes 528 genotyped dogs, out of which 501 had serum fructosamine concentration measured. (JPEG) [file pone.0123173.s002.jpeg]

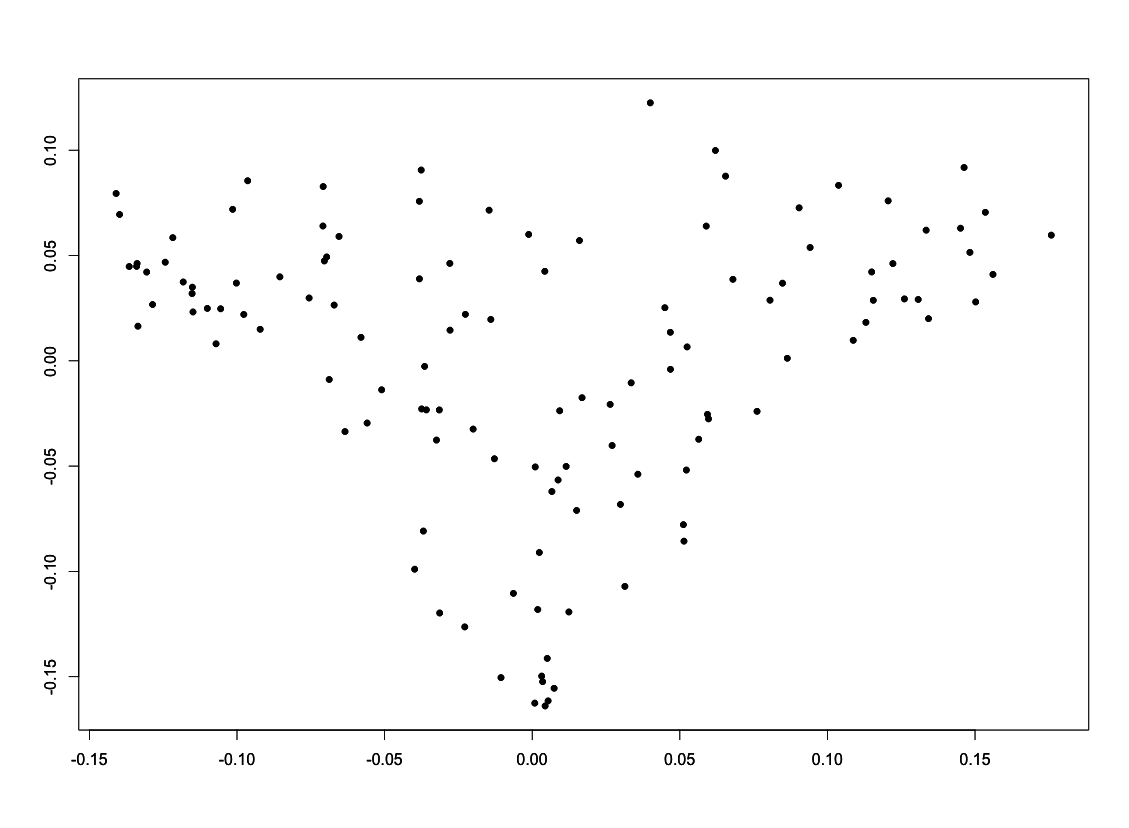

Supplement: S3 Fig — (PNG) [file pone.0123173.s003.png]

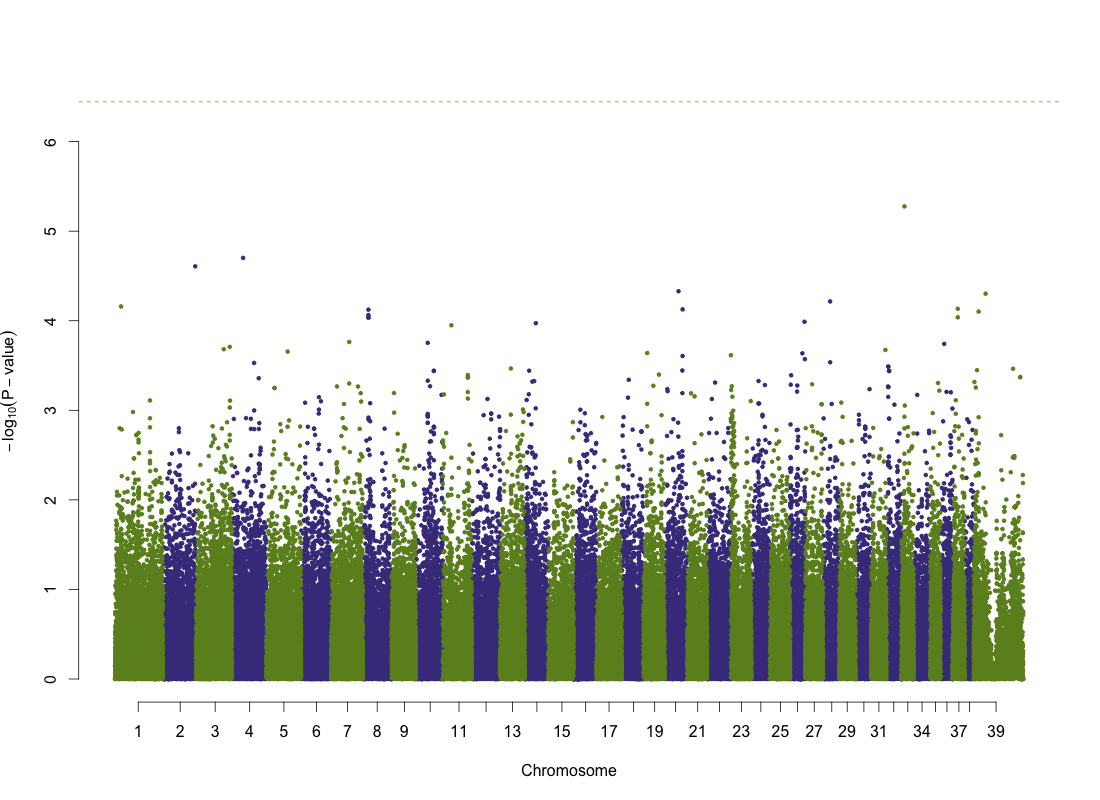

Supplement: S4 Fig — No genome wide significant associations were found in this setup. (PNG) [file pone.0123173.s004.png]

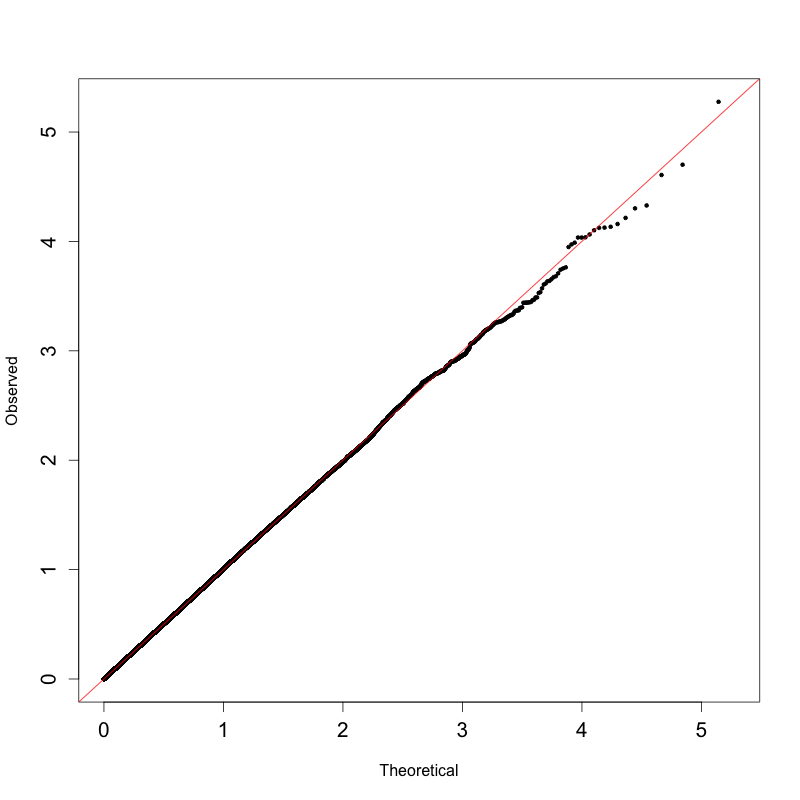

Supplement: S5 Fig — The red line shows the expected profile under the null (slope = 1). (PNG) [file pone.0123173.s005.png]

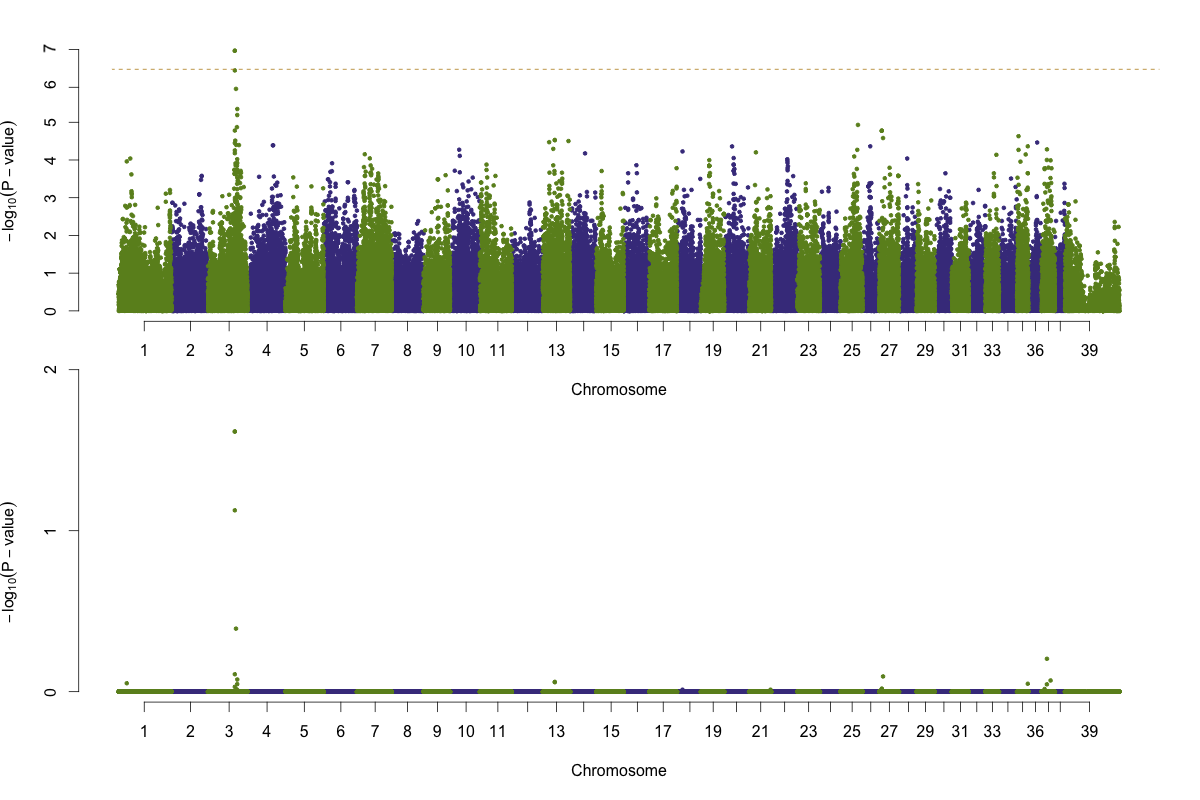

Supplement: S6 Fig — The lower panel shows the p-values from the 10k permutation test, using the GRAMMAR+ transformed mixed model residuals. (PNG) [file pone.0123173.s006.png]

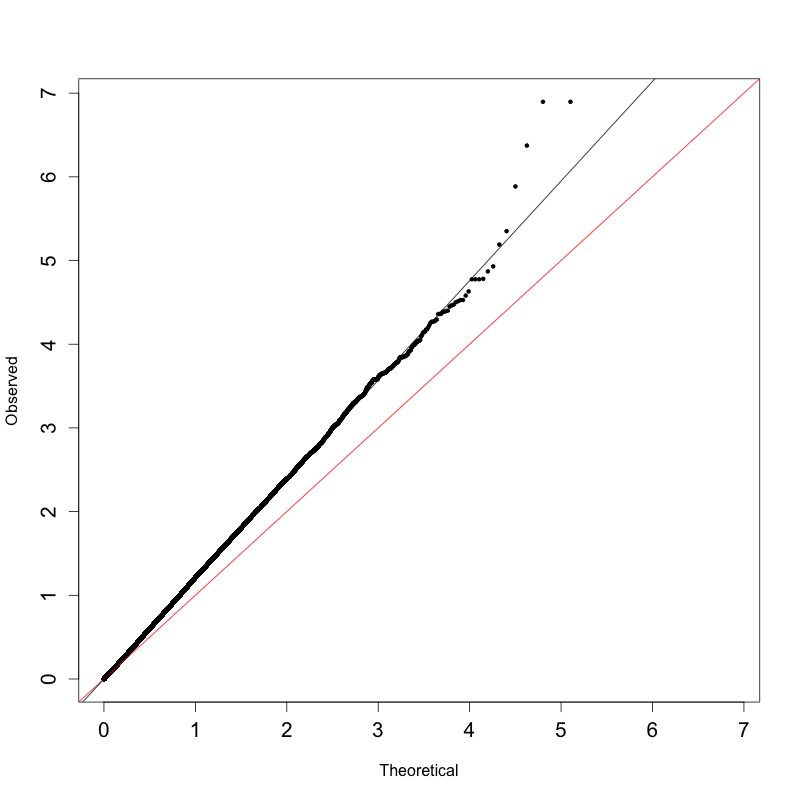

Supplement: S7 Fig — The red line shows the profile expected under the null hypothesis (slope = 1) and the observed profile is indicated by the black line (slope = 1.19). (PNG) [file pone.0123173.s007.png]

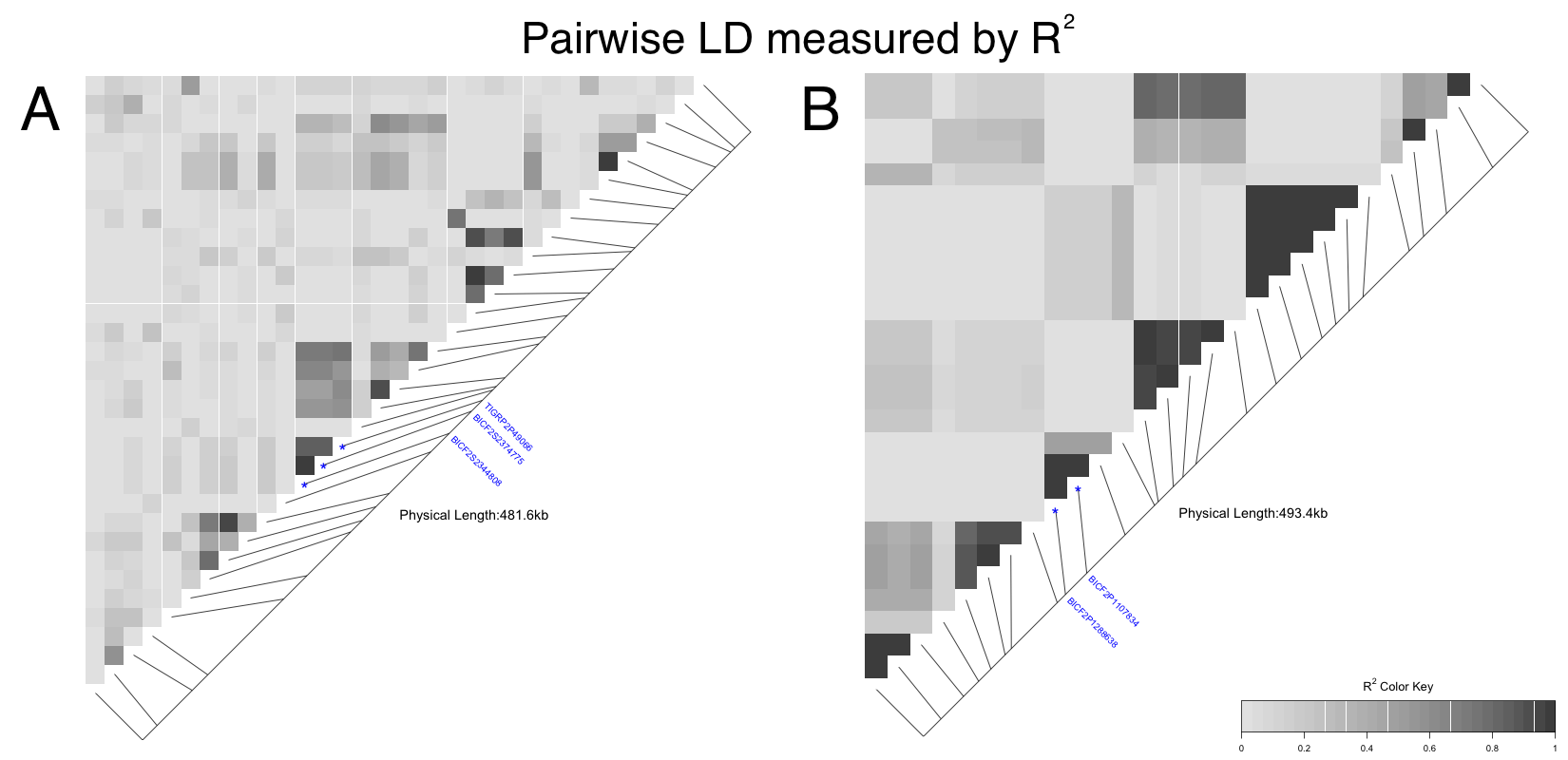

Supplement: S8 Fig — Panel (A) shows a 0.5 Mb segment surrounding the associated region on CFA 3. The leading SNPs in the GWAS analysis are indicated in blue. Panel (B) shows the region of reduced heterozygosity on CFA5. Indicated in blue are the SNPs showing the greatest difference in reference allele count when comparing Belgian and German shepherds vs. remaining breeds. (PNG) [file pone.0123173.s008.png]

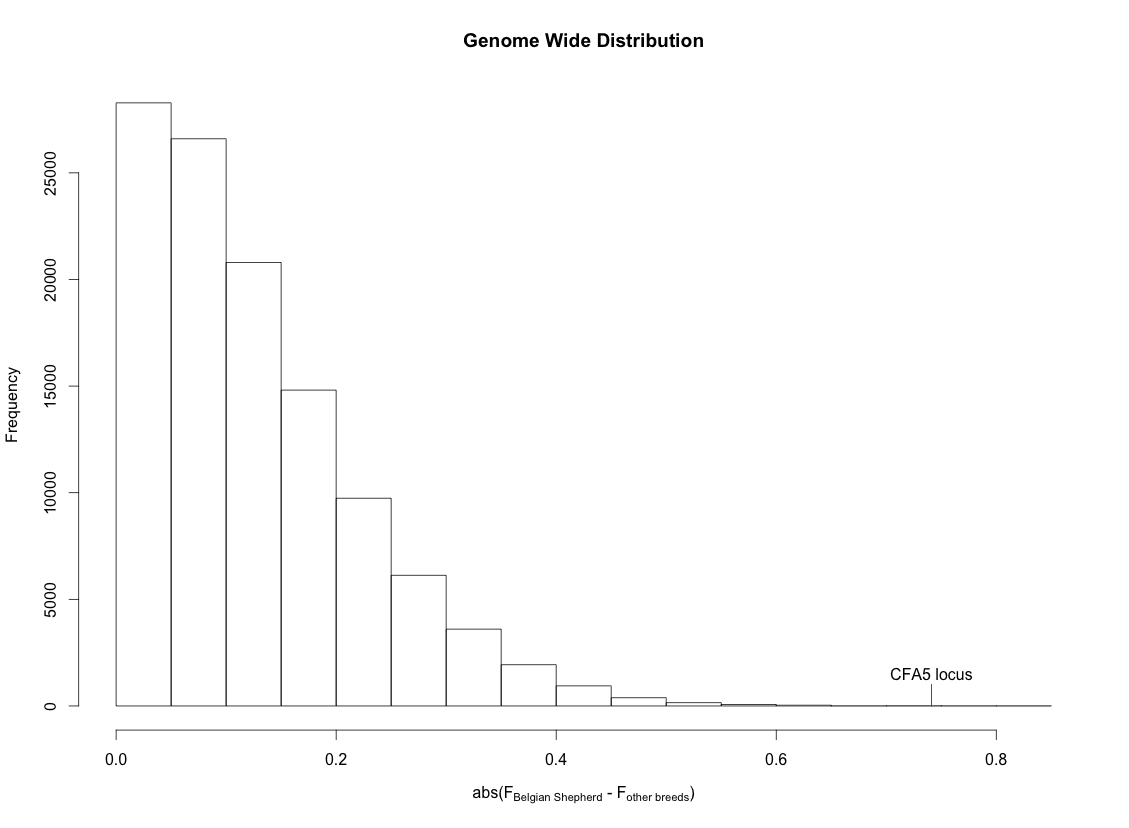

Supplement: S9 Fig — The difference at the leading SNP in the CFA5 locus is highlighted in red. (PNG) [file pone.0123173.s009.png]

$F_{st}$  on CFA 5

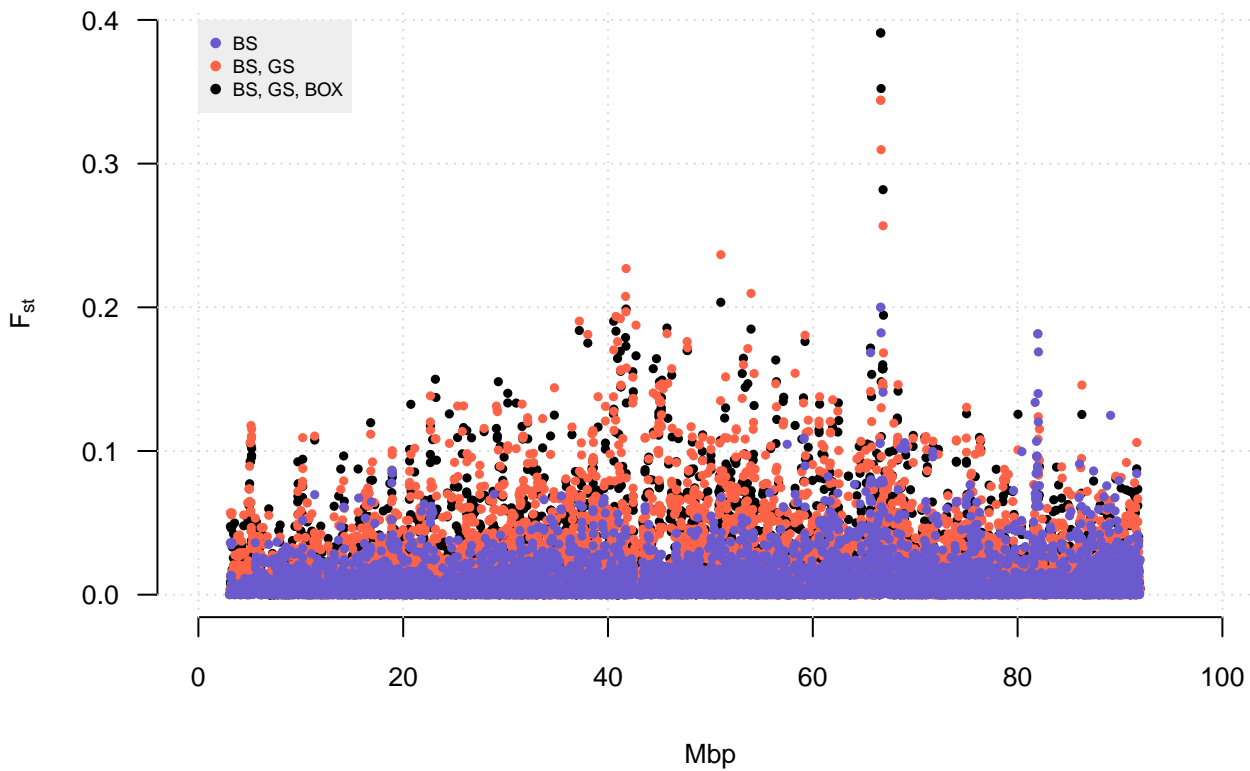

Supplement: S10 Fig — There is a clear divergence between all three breeds (also in all pools) and all other breeds between 66–67Mb. Belgian shepherds alone show also high divergence around 81Mbp. The results are consistent with our analyses of allele frequency differences. (PDF) [file pone.0123173.s010.pdf]
