# Supplementary material for: The Shepherds’ Tale: A Genome-Wide Study across 9 Dog Breeds Implicates Two Loci in the Regulation of Fructosamine Serum Concentration in Belgian Shepherds
Source: PLoS One. 2015 May 13;10(5):e0123173. doi: 10.1371/journal.pone.0123173 (PMC4430432; doi:10.1371/journal.pone.0123173)
Supplement: S1 Table — The table shows the number of markers removed due to low minor allele frequency (row 1) and low call rate (row 2). The first column shows the quality control results when analyzing all breeds together, and successive columns display the results from the breed specific analysis. (PDF) [file pone.0123173.s011.pdf]

Table S1: **Quality control summary.** The table shows the number of markers removed due to low minor allele frequency (row 1) and low call rate (row 2). The first column shows the quality control results when analyzing all breeds together, and successive columns display the results from the breed specific analysis.

	**All breeds**	**Belgian Shepherds**	**German Shepherds**	**Labrador Retrievers**
**Minor allele frequency < 5%**	33,004 (17.8%)	44,433 (24%)	73,030 (39.4%)	55,656 (30%)
**Call rate < 95%**	13,600 (7.3%)	15,600 (8.4%)	13,532 (7.3%)	13391 (7.2%)
**Total**	46,604 (25.1%)	47,774 (32.4%)	74,256 (46.7%)	69,047 (37.2%)
